# Retraction: Wang Y, Yue D. CUL4B promotes aggressive phenotypes of HNSCC via the activation of the Wnt/β‐catenin signaling pathway

**DOI:** 10.1002/cam4.7138

**Published:** 2024-03-28

## Abstract

Wang Y, Yue D. CUL4B promotes aggressive phenotypes of HNSCC via the activation of the Wnt/β‐catenin signaling pathway. Cancer Med. 2019; 8: 2278–2287. https://doi.org/10.1002/cam4.1960

The above article, published online on 18 March 2019, in Wiley Online Library (wileyonlinelibrary.com), has been retracted by agreement between the journal Editor‐in‐Chief, Stephen Tait, and John Wiley and Sons Ltd. Following publication, concerns were raised by third parties regarding Figures 2, 3, and 4. The authors did not respond to requests for the original data. The retraction has been agreed because of concerns that the figures were duplicated, affecting the interpretation of the data and results presented.

Wang Y, Yue D. CUL4B promotes aggressive phenotypes of HNSCC via the activation of the Wnt/β‐catenin signaling pathway. Cancer Med. 2019; 8: 2278–2287. https://doi.org/10.1002/cam4.1960

The above article, published online on 18 March 2019, in Wiley Online Library (wileyonlinelibrary.com), has been retracted by agreement between the journal Editor‐in‐Chief, Stephen Tait, and John Wiley and Sons Ltd. Following publication, concerns were raised by third parties regarding Figures 2, 3, and 4. The authors did not respond to requests for the original data. The retraction has been agreed because of concerns that the figures were duplicated, affecting the interpretation of the data and results presented.

